# Toxic Air Pollutants and Their Effect on Multiple Sclerosis: A Review Study

**DOI:** 10.3389/fpubh.2022.898043

**Published:** 2022-07-06

**Authors:** Mohammad Javad Mohammadi, Kourosh Zarea, Nasser Hatamzadeh, Arash Salahshouri, Asaad Sharhani

**Affiliations:** ^1^Department of Environmental Health Engineering, School of Public Health and Environmental Technologies Research Center, Ahvaz Jundishapur University of Medical Sciences, Ahvaz, Iran; ^2^Air Pollution and Respiratory Diseases Research Center, Ahvaz Jundishapur University of Medical Sciences, Ahvaz, Iran; ^3^Department of Nursing, Nursing Care Research Center in Chronic Diseases, School of Nursing and Midwifery, Ahvaz Jundishapur University of Medical Sciences, Ahvaz, Iran; ^4^Department of Health Promotion and Education, School of Health, Ahvaz Jundishapur University of Medical Sciences, Ahvaz, Iran; ^5^Department of Biostatistics and Epidemiology, School of Health, Ahvaz Jundishapur University of Medical Sciences, Ahvaz, Iran

**Keywords:** multiple sclerosis, brain, toxic air pollutants, chronic diseases, health

## Abstract

Toxic air pollutants are one of the main factors that have the effect of synergism to increase the incidence of multiple sclerosis (MS). This review aims to investigate the effects of toxic air pollutants on the occurrence of multiple sclerosis (MS). A narrative review of the literature was done from 2000 to 2022 based on various databases such as Google Scholar, Web of Science, Springer, PubMed, and Science Direct. In this study, according to the databases, three hundred and sixty articles were retrieved. Of these, 28 studies were screened after review and 14 full-text articles entered into the analysis process. Finally, 9 articles were selected in this study. According to the finding of this study, toxic air pollutants including polycyclic aromatic hydrocarbons (PAHs), heavy metals (HM), volatile organic compounds (VOCs), particulate matter (PM), and gases are the main agents that cause the development and spread of chronic diseases such as respiratory and cardiovascular diseases, chronic obstructive pulmonary disease (COPD), and multiple sclerosis. The result of this study showed that the main sources of emission of toxic air pollutants include industries, cars, power plants, and the excessive consumption of fossil fuels. In general, the inhalation of high concentration of toxic air pollutants can increase the risk of chronic diseases and multiple sclerosis.

## Introduction

Health and environment are related to each other and have interaction effects (1). Both humans and other creatures in some way affect their environment (2, 3). With regards to the advancement of technology and its incompatibility with the environment and the change of human civilization during different eras, ignoring the correct relationship between human needs and the environment has made the life of creatures on earth more complex (4, 5). The main sources of emission and production of toxic air pollutants are human's activities such as industrial processes, agriculture, activities of power plants (man-made origin), road traffic, combustion of fuel (petroleum, oil, gasoline, diesel, wood, coal), pollutants enter the oceans through evaporation, volcanic eruptions (natural), and forest fires (6–8).

Toxic air pollutants can cause dysfunction and destroy the functions of the cardiovascular, respiratory, circulatory, reproductive, and nervous systems (9–11). Also, these pollutants can cause dangerous diseases such as cancers of the lungs, heart, liver, blood, kidney, bladder, and brain (9–11). This pollution has unpleasant effects on the health of humans and other organisms (12, 13). One of the most important effects of dangerous air pollutants is the effect on multiple sclerosis (MS) (14). Multiple sclerosis is known as a disease of the modern and industrial world, but this does not mean that no one has had MS before. Even in previous years there were patients, but with the advent of the modern world, the disease has become more severe (15, 16).

This means that in the last 20 years, we have been facing an abundance of diseases that are due to the industrialization or modernization of society, and the incidence of MS is increasing (17, 18). The cause of MS is still unknown (19). Multiple sclerosis is exacerbated by factors such as air pollution, vitamin D deficiency, environmental pollution, severe stress, and traffic (20, 21). The risk of developing MS in metropolitan areas due to air pollution is higher than in other areas (22, 23). There are several factors involved in this upward trend, including the industrialization of society (24). Smoking or hookah, vitamin D deficiency, and air pollution are other factors that increase the incidence of multiple sclerosis (25, 26).

In families with a history of MS, the risk of other family members being infected is about 10%, which indicates that environmental factors are involved in the development of multiple sclerosis (27). Multiple sclerosis is not curable like many diseases, but if treated, it can be controlled and the patient can lead a relatively normal life (28, 29).

In the world that has between 2.5 and 3 million people with MS (22), the latest statistics provided in the country show that about 70,000 Iranians also suffer from multiple sclerosis (30). In urban areas, the risk of developing multiple sclerosis is higher than in rural areas because of the high concentration of air pollutants and long-term exposure to toxic pollutants (31). The prevalence of MS in areas with low-level of air pollutants is much lower, which indicates that air pollution can be one of the high-risk factors for MS (32).

Considering the importance of neurological systems' dysfunction caused by exposure to air pollutants, the aim of this narrative review was the evaluation of toxic air pollutants and their effect on multiple sclerosis.

## Methods

### Eligibility Criteria and Search Strategy

This narrative review study was conducted on references available in various databases: Science Direct, PubMed, Web of Science, Springer, and Google Scholar (Table 1). Years of publication 2000–2022 and English language were the main criteria for search limitation. Three hundred and sixty articles according to databases were retrieved.

**Table 1:** Search terms and query results.

**Term**	**Google Scholar**	**Web of Science**	**Springer**	**Science Direct**	**PubMed**	**Unique results**
Multiple sclerosis	52	23	11	18	12	116
Toxic air pollutants and Multiple sclerosis	41	19	13	15	7	95
Health endpoint	26	14	9	11	6	66
Toxic air pollutants	21	13	5	6	4	49
Toxic air pollutants and Brain	10	8	3	5	8	34
Total	150	77	41	55	37	360

### Collection Data

“Multiple sclerosis,” “toxic air pollutants and multiple sclerosis,” “health endpoint,” “toxic air pollutants” and “toxic air pollutants and brain” were terms that were used in the first stage search.

Studies were prepared based on searches of 150 articles in the Google Scholar, Web of Sciences received 77 articles, Springer database 41 articles, Science Direct 55 articles, and PubMed database 37 articles. Review time efficiency of studies was limited to the range of 2000–2022.

**Figure 1:**
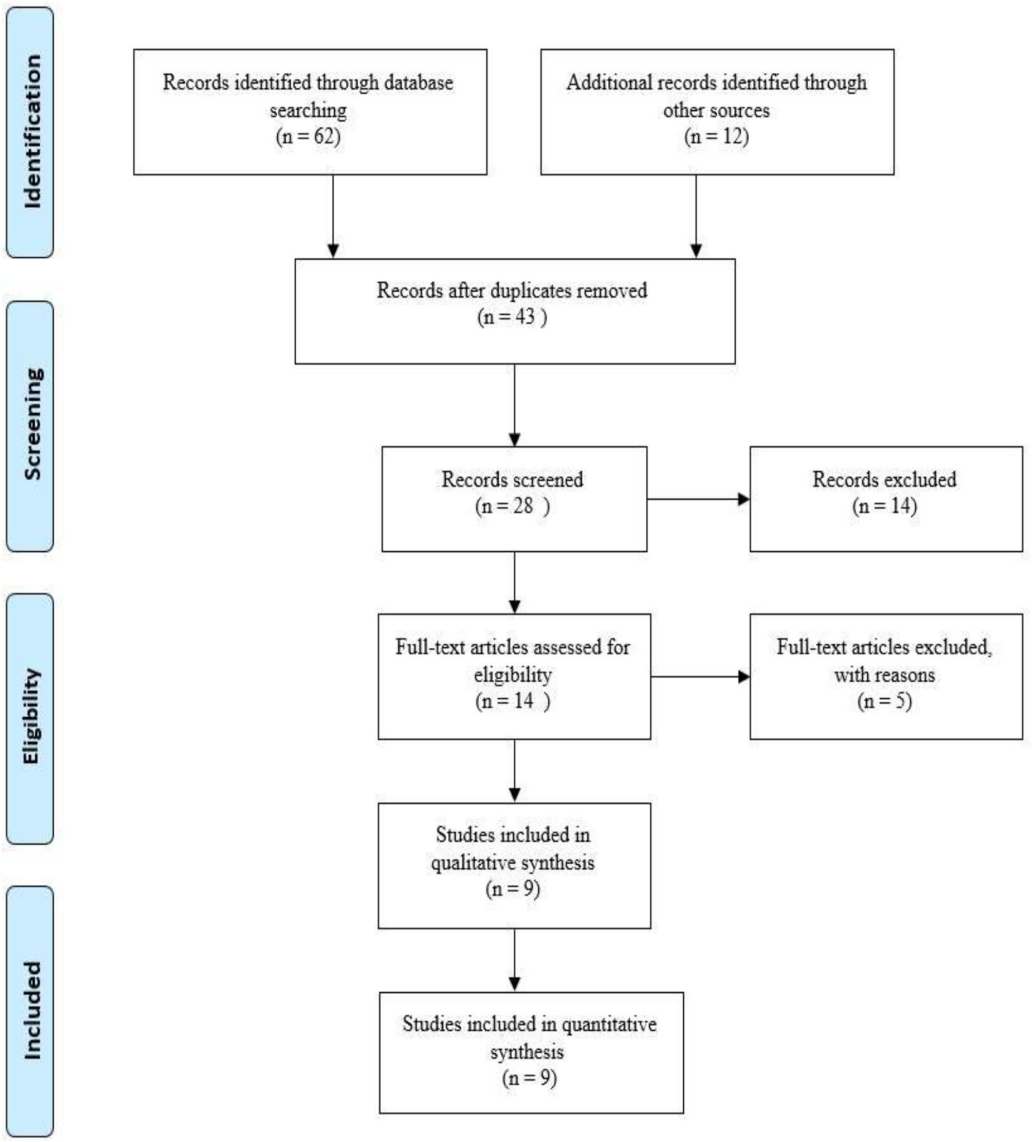
Representation of the search strategy based on PRISMA flow diagram.

A total of 62 and 14 articles were found and selected based on records identified through database searching, and additional records were identified through other sources. In the next stage, 28 studies were screened after review and 14 full-text articles were entered into the analysis process. Finally, 9 articles were selected for this study. How to prepare studies and the selection process of articles based on the PRISMA flow diagram is shown in Figure 1.

### Ethical Approval

Ethical issues (including plagiarism, misconduct, data fabrication, falsification, submission, double publication, and redundancy) have been completely observed by the authors. The Ethics License of the present study was acquired from the Ethics Committee of Ahvaz Jundishapur University of Medical Sciences (Code of ethics: IR.AJUMS.REC.1400.708).

## Results And Discussion

### Multiple Sclerosis (MS)

Nowadays, the main autoimmune inflammatory and degenerative disorder of the central nervous system that has clinical outcomes which are extremely variable is multiple sclerosis (16). Multiple sclerosis is a chronic autoimmune disease in which the immune system attacks the nerve tissue and destroys fat around the nerves (myelin) (33). In multiple sclerosis, the myelin of the central nerves (brain and spinal cord) becomes inflamed and damaged in the form of individual or large plaques of small or large size (34). Myelin can be compared to the insulation coating on an electrical wire (35). When myelin is damaged and nerve fiber is exposed, the messages that travel along that nerve may slow down or stop, failing to transmit the message properly (36). In this case, the brain loses control of its organs. In the central nervous system, symptoms are very different depending on where there are plaques (37). The age range for multiple sclerosis is between 20 and 50 years, and in recent years the incidence of the disease in children and people over 50 years has increased sharply (38, 39). MS is more prevalent in women, with a ratio of 3 to 4 women to one man (40).

Sex hormones are involved in the disease process since the sex hormones cause the disease to spread in women. Most people with multiple sclerosis have a relapsing–remitting illness (41). Patients with multiple sclerosis experience periods of new symptoms or recurrences that occur over days or weeks and usually improve partially or completely. Following these recurrences, there are periods of recovery that can last for months or even years (42, 43).

Low body temperature can temporarily worsen the signs and symptoms of MS, but is not considered a recurrence (44). About 60 to 70% of patients with relapsing–remitting MS eventually develop symptoms that progress with or without periods of recovery that is known as secondary progressive MS (45, 46). Worsening symptoms usually include movement and walking problems (47). The rate of disease progression varies among patients with progressive secondary MS (46). Some people with MS experience a gradual onset and continuous progression of symptoms without any recurrence (42). These patients are known to have primary progressive MS (48).

The diagnosis of multiple sclerosis is often based on the rejection of other diagnoses that may cause similar signs and symptoms, which are called differential diagnoses (49, 50). A person with inflammation has all the neurological signs or symptoms (51). The most common of these symptoms are autonomic, visual, motor, and sensory problems (52).

**Figure 2:**
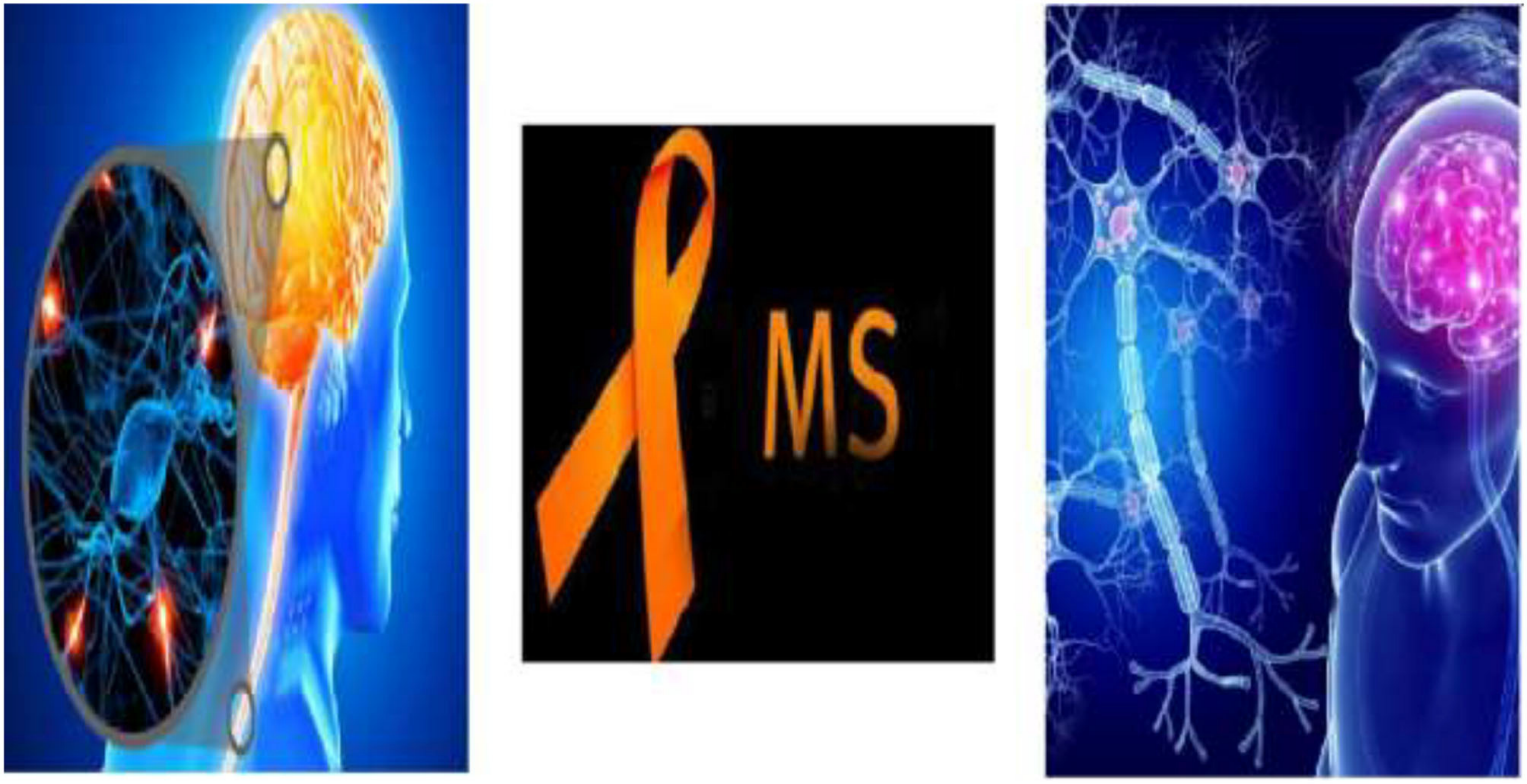
Immune system attacks the nerve tissue (multiple sclerosis).

The main specific symptoms among people with multiple sclerosis include lethargy or drowsiness such as tingling, spasm, muscle weakness, involuntary reactions, muscle cramps or inability to move, inability to coordinate and balance muscle coordination, difficulty urinating and defecating, tiredness, difficulty speaking or dysphasia, visual problems (eyeball movement, blurred vision, or diplopia), severe or chronic pain, difficulty to think, and depression and emotional distress (53–56). Figure 2 shows the route of the central nerves system after the immune system attacks the nerve tissue.

### Air Pollutants

Air pollution occurs when large volumes of harmful particles such as gases, particles, and biomolecules enter the Earth's atmosphere (57). Air pollution is a mixture of suspended particles and gases whose concentration has reached a range harmful to humans.

Growth population, emissions of greenhouse gases, destroying vegetation and forests and cutting down trees indiscriminately, excessive consumerism of the world, excessive production of carbon dioxide and global warming, increasing industrialization, and excessive use of fossil fuels are the most important causes for the release of toxic air pollutants, their entry into the food cycle (plants and animals), and consumption by people (58, 59).

Air pollution results in several major causes of death in the world including stroke, heart disease, lung cancer, and respiratory diseases (COPD, bronchitis and asthma) (60, 61). Air pollution in various ways can have long-term and short-term harmful effects on human health (62). The impact of air pollution on people is different because some people are much more vulnerable to air pollution (63). Younger children and the elderly are more likely to be affected by air pollution (64). The extent of damage usually depends on the time exposed to toxic air pollutants and the concentration of toxic pollutants (62, 64).

**Figure 3:**
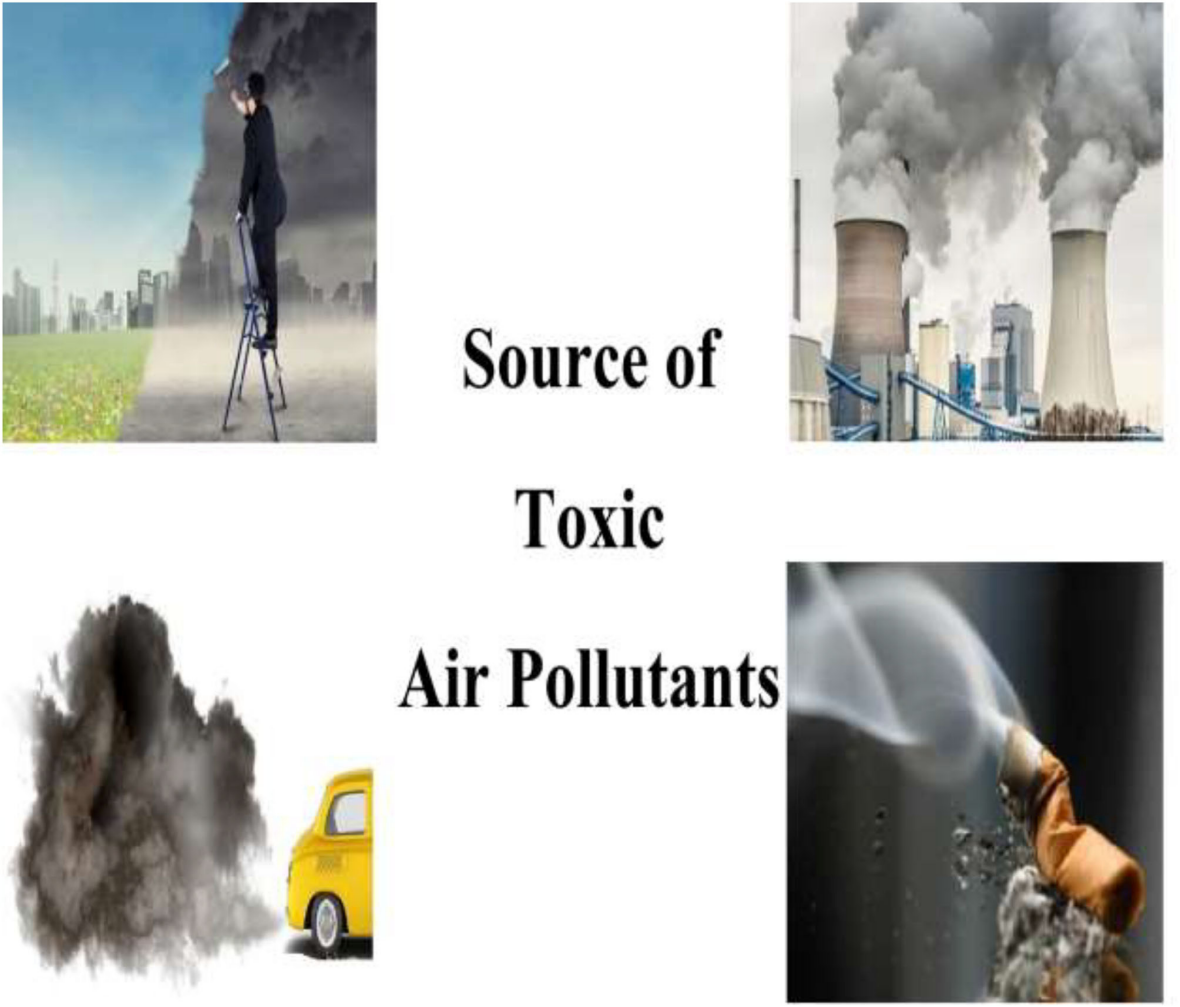
Sources of emission of toxic air pollutants in the environment.

Toxic air pollutants through the lungs, skin, mucous membranes, mucous membranes of the eyes, ears, nose, and gastrointestinal tract enter the body and destroy the body's cells (65). Air pollution is associated with behavioral changes because because it causes people to spend less time in the open and lead a more sedentary lifestyle, which can cause psychological distress and social isolation (66, 67). The main negative effects of air pollution on the nervous system are more irritability, aggression, anger, and intolerance (68, 69). Figure 3 shows sources of emission of toxic air pollutants in the environment.

### Toxic Air Pollutants and Effects on Multiple Sclerosis

Toxic air pollutants (polycyclic aromatic hydrocarbons (PAHs), BTEX (benzene, toluene, ethylbenzene, and xylene), heavy metals, VOCs, and particulate matter) through the bloodstream reach the brain and can directly damage the neurons (58, 70–72).

Toxic air pollutants get into the body mainly through breathing. They can also be ingested (e. g., children eating soil contaminated with lead) or absorbed through the skin (73, 74).

Inhalation of toxic air pollutants (breathing), swallowing, or ingestion of foods infected with toxic air pollutants (eating foods) and dermal contact with heavy metal and polycyclic aromatic hydrocarbons (skin) are the main routes of entire toxic air pollutants and destruction on human (75, 76). Air pollutants can cause extensive damage by disrupting the regulation of microglia and immune cells in the brain. Microglia may confuse intruders with pathogens and release chemicals to kill them (77, 78). These chemicals can accumulate and cause inflammation, and chronic inflammation of the brain is involved in the destruction of nerve cells (79, 80).

According to the reports of different studies, toxic air pollutants can slip through the plasma membrane of the alveoli and small air sacs in the lungs, collect through the capillaries, and then the pollutants are distributed in the blood throughout the body (77). Although some of these pollutants may eventually break down the blood–brain barrier, there is no need for a contaminant to enter the brain to cause a problem even though the immune system can also react to particles in the lungs or bloodstream, causing widespread inflammation that affects the brain (81).

A swallowed particle can have indirect neurological effects through the intestine (82). Researchers are now recognizing strong links between the gut microbiome and the brain, and studies show that delivering small particles to the gut can cause systemic inflammation (83, 84). Exposure to toxic air pollutants is known to be an environmental risk factor that causes brain aging (85, 86). The risk of dementia and Alzheimer's disease increases with exposure to toxic air pollutants (87).

Nitrogen dioxide (NO_2_) and hydrocarbons (HC) are produced by the combustion of vehicle fuels (88, 89). NO_2_ and HC increase the risk of developing autism (90, 91). Short- or long-term exposure to air pollution is also associated with an increased risk of stroke and acute coronary syndrome, such as heart attack (92–94). Exposure to toxic air pollutants is directly related to the underlying clinical diseases of stroke (systemic inflammation, oxidative stress, atherosclerosis, thrombosis, and arrhythmia) (95–97).

**Figure 4:**
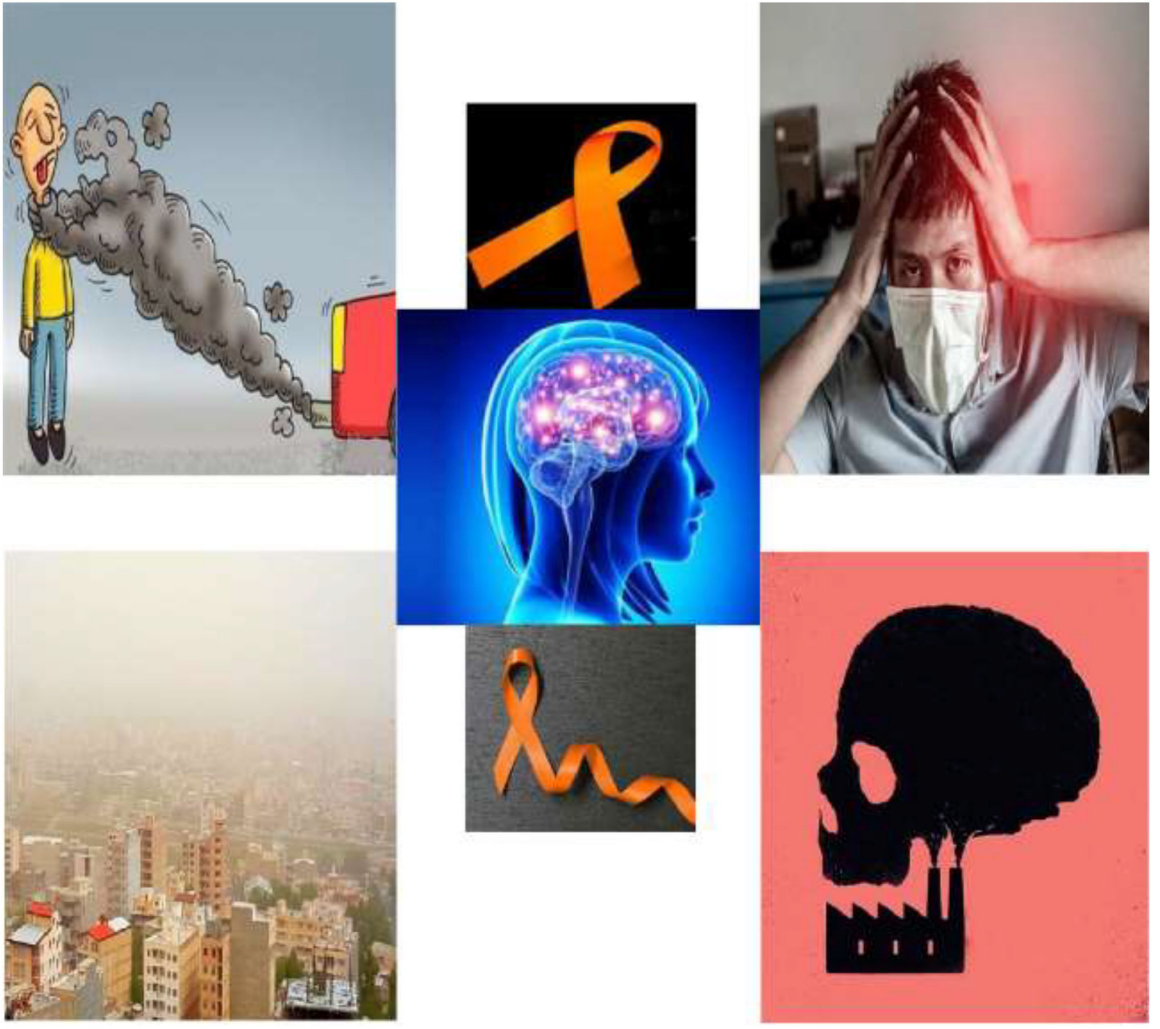
Sources of toxic air pollutants and its effects on multiple sclerosis.

Multiple sclerosis has a variety of causes and some environmental factors such as air pollution can trigger an abnormal immune response (98, 99). Air pollution can also be directly linked to affect neurological diseases such as Alzheimer's and Parkinson's (100, 101). Inhalable toxic air pollutants can increase the risk of developing MS (102, 103). Since MS is an inflammatory disease, air pollution can affect it and increase MS attacks. Smoking is one of the main agents that as an environmental factorplays a main role in increasing the risk of prevalence multiple sclerosis (104–106). Based on the results of different studies, the risk of the incidence multiple sclerosis has a direct relationship with cigarette smoking because of the presence of high amounts of cadmium that affect the susceptibility to MS (104, 107, 108). Seasonal changes in the condition of patients with MS show that climate change and air pollution can be effective in relapsing MS. The toxic air pollutants are absorbed into the lungs through the respiratory tract, causing systemic pulmonary inflammation and activation of cells that then secrete proinflammatory cytokines in the brain. Second, toxic air pollutants are absorbed directly through the olfactory bulb and may cause inflammatory reactions in the brain, and third, genetic mutations caused by toxic air pollutants affect its onset (109–113). Figure 4 shows sources of toxic air pollutants and its effects on multiple sclerosis.

Controlling the level of vitamin D in the body using cod liver oil, cheese, yogurt, butter, milk; regular use of prescription drugs; avoiding negative thoughts; reducing daily stress; exercises such as yoga, swimming, and walking; avoiding smoking; consumption of fish; coffee consumption; maintaining a healthy body mass index (BMI); and living in places with very low levels of air pollution are among the most important measures that can help prevent multiple sclerosis.

## Conclusion

In this study, we investigated toxic air pollutants and their effect on the increased prevalence of multiple sclerosis. The results showed that the inhalation and exposure to air pollution such as polycyclic aromatic hydrocarbons, heavy metals, volatile organic compounds, and particulate matter were related to the actual risks of different systems and organs of the body such as the brain, lungs, and heart.

The finding this study showed is that toxic air pollutants can have synergistic and aggravating properties in increasing cases of multiple sclerosis. It should be noted that according to health organizations, such as World Health Organization (WHO), the trend incidence and severity of recurrent MS attacks have been increasing. Increase acknowledge of the relationship between toxic air pollutants on multiple sclerosis are the main applications of the results in this study.

Also, based on reports to better understand and more knowledge of the characteristics of toxic air pollutants can be a great help to health managers and policymakers in taking preventive and protective measures. An increase in the number of air monitoring stations, preparation and approval of government regulations related to reducing air pollutant emissions, and reducing the use of these fossil fuels are the most important activities that can causes reduces emissions and have a significant effect on decreasing the health endpoint of toxic air pollutants.

In general, further studies are vital and required to determine the effects of toxic air pollutants on all body systems (respiratory, cardiac, vascular, gastrointestinal, nervous, circulatory, urinary, and reproductive) and body organs (brain, heart, kidney, lung and liver).

## Author Contributions

MM, KZ, NH, ASa, and ASh were principal investigators and advisors of the study, drafted the manuscript, and performed the statistical analysis. All authors contributed to the design, data analysis, assisted in the preparation of the final version of the manuscript, read, and approved the final version of the manuscript.

## Funding

This study was funded by the Ahvaz Jundishapur University of Medical Sciences.

## Conflict of Interest

The authors declare that the research was conducted in the absence of any commercial or financial relationships that could be construed as a potential conflict of interest.

## Publisher's Note

All claims expressed in this article are solely those of the authors and do not necessarily represent those of their affiliated organizations, or those of the publisher, the editors and the reviewers. Any product that may be evaluated in this article, or claim that may be made by its manufacturer, is not guaranteed or endorsed by the publisher.
